# Genomic and epidemiological report of the recombinant XJ lineage SARS-CoV-2 variant, detected in northern Finland, January 2022

**DOI:** 10.2807/1560-7917.ES.2022.27.16.2200257

**Published:** 2022-04-21

**Authors:** Erika Lindh, Teemu Smura, Soile Blomqvist, Kirsi Liitsola, Hanna Vauhkonen, Laura Savolainen, Jaana Ikonen, Jukka Ronkainen, Jyri Taskila, Tea Taskila, Pertti Sakaranaho, Carita Savolainen-Kopra, Olli Vapalahti, Niina Ikonen

**Affiliations:** 1 Department of Health Security, Finnish Institute for Health and Welfare (THL), Helsinki, Finland; 2 Department of Virology, Faculty of Medicine, University of Helsinki, Helsinki, Finland; 3 HUS Diagnostic Center, HUSLAB, Clinical Microbiology, Helsinki University Hospital, Finland; 4 Northern Finland Laboratory Center Nordlab, Oulu, Finland; 5 Northern Finland Laboratory Center, Nordlab, Länsi-Pohja, Finland; 6 Primary Health Care Center City of Tornio, Finland and Center for Life Course Health Research, University of Oulu, Finland; 7 Länsi-Pohja Health Care District, Kemi, Finland; 8 Primary Health Care Center City of Kemi, Kemi, Finland; 9 Municipality of Keminmaa, Keminmaa, Finland; 10 Department of Veterinary Biosciences, Faculty of Veterinary Medicine, University of Helsinki, Helsinki, Finland

**Keywords:** SARS-CoV-2, variant, recombinant, omicron, XJ-lineage

## Abstract

Recombinant sequences of the SARS-CoV-2 Omicron variant were detected in surveillance samples collected in north-western Finland in January 2022. We detected 191 samples with an identical genome arrangement in weeks 3 to 11, indicating sustained community transmission. The recombinant lineage has a 5’-end of BA.1, a recombination breakpoint between *orf1a* and *orf1b* (nucleotide position 13,296–15,240) and a 3’-end of BA.2 including the *S* gene. We describe the available genomic and epidemiological data about this currently circulating recombinant XJ lineage.

Ten suspected recombinant sequences were initially detected by the national genomic surveillance of severe acute respiratory syndrome coronavirus 2 (SARS-CoV-2) in Finland in surveillance samples collected in January 2022 (weeks 3 and 4) in the Länsi-Pohja hospital district in north-western Finland. The Länsi-Pohja hospital district serves six small municipalities with a total population of 59,000, located near the border to Sweden. As inhabitants of the border municipalities frequently move between the two countries, there was a risk of cross-border spread. Intensified surveillance revealed at least 191 domestic cases, and database search retrieved five international cases by mid-March 2022 (week 11), indicating sustained community transmission and leading to Pango designation (Phylogenetic Assignment of Named Global Outbreak) of the XJ recombinant lineage.

We provide here a description of the confirmation and genomic characterisation of the novel XJ recombinant, describe the early cases and transmission events and discuss the available epidemiological data.

## Genomic investigation

**Figure 1:**
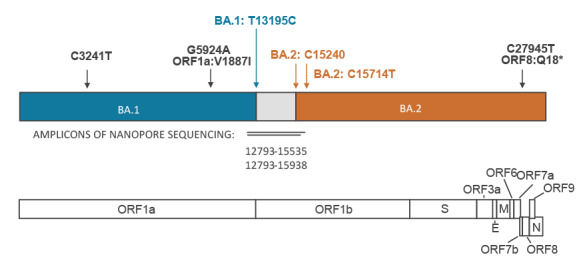
Schematic representation of the SARS-CoV-2 BA.1/BA.2 recombinant genome arrangement

Recombinant SARS-CoV-2 genome sequences were observed in independent Illumina sequencing runs of surveillance samples in 2022 from week 3 onwards (sequencing and sequence analyses were conducted as described in [1-3]). Sequence analysis suggested a recombination breakpoint between nucleotide positions 13,296 and 15,240. The recombination was confirmed by long-read sequencing (Oxford Nanopore Technology) of two PCR amplicons spanning the recombination breakpoint (Figure 1).

SARS-CoV-2: severe acute respiratory syndrome coronavirus 2.

The recombinant genomes contain all lineage-defining mutations of BA.1 until the synonymous T13195C mutation at the 3’end of the ORF1a coding region (blue), while they lack the BA.1-specific C15240T mutation in ORF1b coding region, and contain all BA.2 lineage-defining mutations starting from C15714T in ORF1b (red), suggesting a recombination breakpoint between nucleotides 13,195 and 15,240. The recombination was confirmed by long-read nanopore sequencing of two PCR amplicons spanning the recombination breakpoint (forward primer 12793-GGAGGTAGGTTTGTACTTGCACTG-12817 and either one of two reverse primers 15558-TGCATTAACATTGGCCGTGACA-15536 or 15960-GGCCCCTAGGATTCTTGATGGA-15938). The sequencing library was prepared using the Nanopore SQK-LSK110 kit and the sequencing was carried out using a MinION R9.4.1 flowcell.

**Figure 2:**
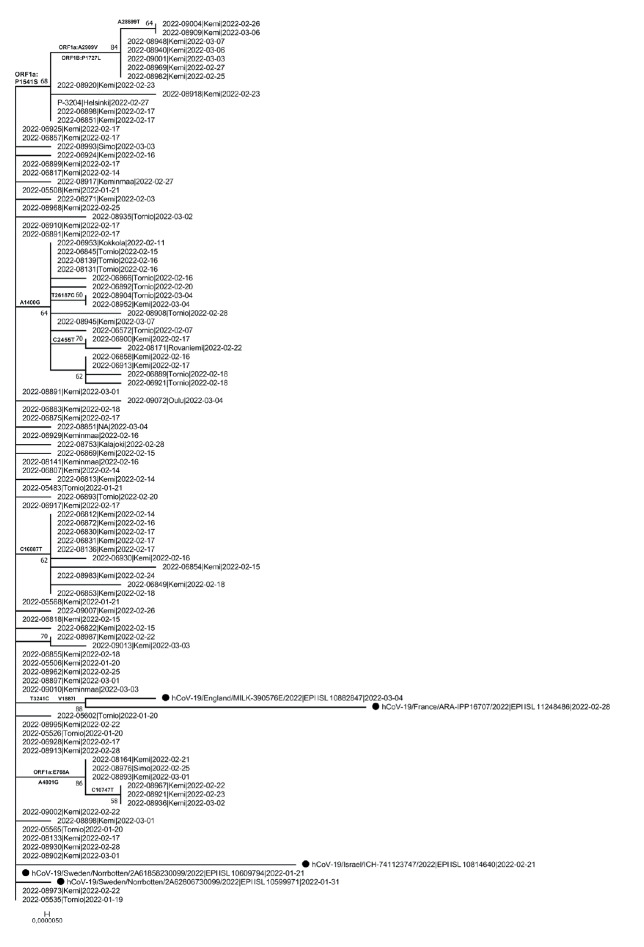
Phylogenetic trees of all SARS-CoV-2 lineage XJ recombinant genomes that had fewer than 1,000 ambiguous nucleotides, Finland, January–February 2022 (n = 96)

Ninety-six of 106 XJ recombinants with nearly complete genome sequences (fewer than 1,000 ambiguous nucleotides) were included in the phylogenetic analysis. The analysis suggested that 33 of the 96 Finnish lineage XJ genomes, including the earliest sequences, were identical to each other, while the sequences sampled later tended to have more diversity (Figure 2).

SARS-CoV-2: severe acute respiratory syndrome coronavirus 2.

The genomes were aligned using Nextalign [11] and the tree was inferred using maximum likelihood method implemented in MEGA6 software. Clade-specific mutations are shown in each branch. Sequences detected outside Finland are marked with filled circles.

**Figure 3:**
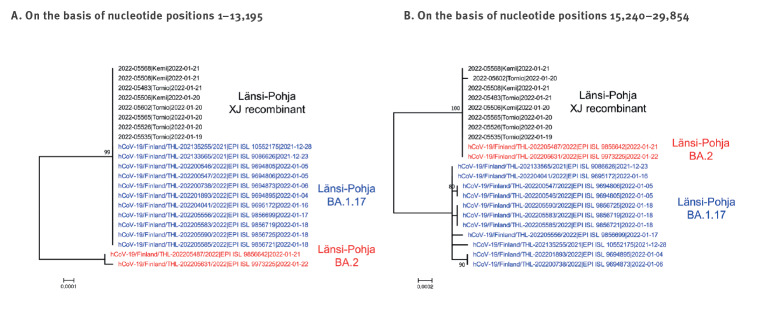
Potential parental genomes of SARS-CoV-2 lineage XJ recombinants, Länsi-Pohja district, Finland, January–February 2022 (n = 19)

In order to identify the potential parental sequences for the recombinant genomes, we compared the genome regions flanking the recombination breakpoint with all 2,338 BA.1 and BA.2 sequences available in the GISAID database collected until 31 Jan 2022 from Finland. We detected 23 BA.1.17 and two BA.2 sequences identical to, respectively, the 5’ and 3’ ends of the recombinant genome. The BA.1.17 sequences had two signature mutations, C3241T and G5924A (ORF1a:V1887I), while the BA.2 sequences had one, C27945T (ORF8:Q18*), introducing a premature stop codon and resulting in a truncated ORF8 protein. Notably, both BA.2 and 11 of the 23 BA.1.17 sequences observed in the national genomic surveillance originated from the relatively small Länsi-Pohja hospital district, representing two of the nine BA.2 and 11 of the 571 BA.1 samples collected in the area during weeks 1 to 4 in 2022 (Figure 3). Using BLAST, we detected identical sequences for the 5’ and the 3’ ends flanking the recombination breakpoint in globally sequenced samples.

SARS-CoV-2: severe acute respiratory syndrome coronavirus 2.

The genomes were aligned using Nextalign [11] and the tree was inferred using maximum likelihood method implemented in MEGA6 software. The potential parental genomes are shown in blue for BA.1.17 and red for BA.2. For the sake of clarity, only the earliest sequences of the recombinant lineage XJ (black) and those of the potential parents (lineage BA.1.17 (blue) and BA.2 (red)) detected in Länsi-Pohja hospital district were included in the analysis.

## Identification of early cases and transmission events

We traced the first 10 cases of infection with SARS-CoV-2 lineage XJ detected in the country in weeks 3 and 4 of 2022 and established epidemiological links between them. Five cases worked or studied at the same educational unit. Two cases lived in the same apartment building, one of whom attended a kindergarten that was later linked to two family clusters. The sampling date of the first cases fell within a range of four days in January 2022, therefore none of the identified cases were considered as an index case.

The median age of all 191 confirmed cases with XJ detected in Finland between week 3 and 11 was 42 years (range: 0–93). Data on COVID-19 vaccination status and clinical data were available for 43 confirmed cases with XJ. Of these, 35 had received at least two doses of COVID-19 vaccine, with the latest dose administered within 6 months before confirmed infection. Twenty-seven had received Comirnaty (BioNTech-Pfizer), three Spikevax (Moderna) and five had received a combination of Comirnaty and Spikevax, Comirnaty and Vaxzevria (Astra Zeneca) or Spikevax and Vaxzevria. No remarkable differences in the clinical course of disease have been identified so far. One death was recorded in an elderly individual with underlying disease.

## Distribution of the recombinant

**Figure 4:**
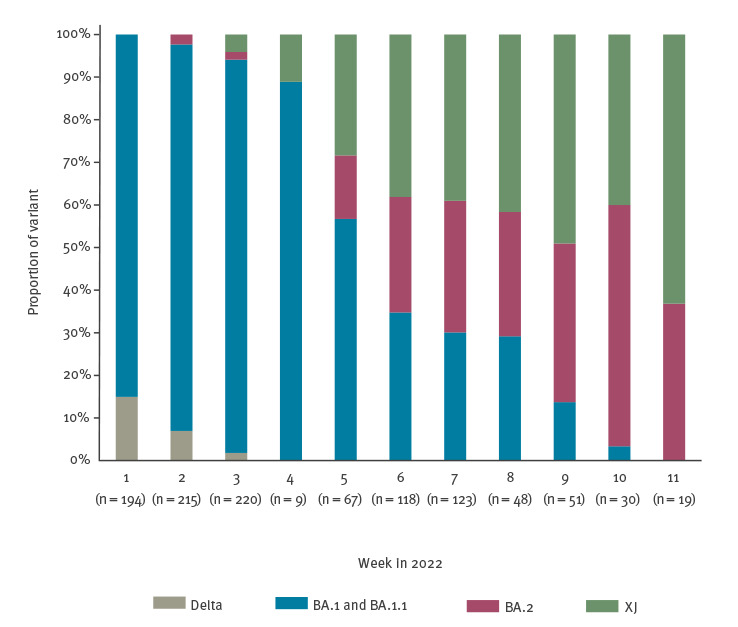
SARS-CoV-2 variant distribution, Länsi-Pohja hospital district, Finland, week 1–11/2022 (n = 191 XJ among 1,094 sequences)

In response to the initial identification of the recombinant sequences from week 3/2022 onwards, and because the tracing of early cases and transmission events suggested community transmission, genomic surveillance of variants was intensified in the affected area in week 7. The number of sequenced samples was increased to detect as many cases as possible and to monitor changes in the prevalence of the XJ variant with increased sensitivity [4]. A large number of recombinant sequences in the Länsi-Pohja hospital district was observed in week 7 and onwards. Forty-eight cases were detected in week 7, representing 39% of the SARS-CoV-2 samples sequenced in that week (Figure 4). By week 11, 191 recombinant cases were confirmed through sequencing, the great majority of which were from Länsi-Pohja hospital district. Single cases were subsequently also detected in surveillance samples from several cities across the country including Helsinki, the capital. Based on sequences available in the GISAID database, XJ sequences were further identified in Sweden (n = 2), the United Kingdom (n = 2), France (n = 1) and Israel (n = 1) (Figure 2). A sample collection date as early as week 3/2022 was recorded for a sample taken in a Swedish town bordering Länsi-Pohja, indicating that cross-border spread may have occurred at an early stage.

SARS-CoV-2: severe acute respiratory syndrome coronavirus 2.

## Discussion 

Rapid changes in the global SARS-CoV-2 variant composition have occurred several times during the coronavirus disease (COVID-19) pandemic since its first emergence in the end of 2019 [5,6]. The SARS-CoV-2 variant Omicron (Pango lineage designation B.1.1.529) emerged in late 2021, displacing the dominant Delta variant (B.1.617.2) in most parts of the world within a few weeks [7,8]. Recombination is a widespread evolutionary mechanism among viruses, including coronaviruses, giving rise to genomic diversity independent of mutations. It has been associated with diverse phenotypical changes including expansion of the viral host range, reviewed in [9]. With several coronavirus lineages circulating in the population, recombinants are likely to emerge and are now increasingly reported from different countries [10]. 

The national genomic surveillance of SARS-CoV-2 in Finland was initiated jointly by the National Institute for Health and Welfare (THL), the University of Helsinki and the Institute for Molecular Medicine Finland (FIMM) in the end of 2020 to support the timely detection of circulating variants and the global effort to monitor and assess the evolution of SARS-CoV-2. In addition, three other clinical laboratories, TYKS Laboratoriot, FIMLAB and Vita Laboratoriot, participate in the national genomic surveillance of SARS-CoV-2 in Finland. The XJ recombinant described here was detected through the national surveillance. It has arisen from BA.1 and BA.2 parental lineages and harbours the S gene of the widely circulating BA.2 lineage without any novel mutations. Since no changes to the receptor binding region, recognised antibody binding sites or sites involved in the proteolytic cleavage of the spike protein have been introduced in the XJ recombinant, it is plausible that it will display phenotypical properties similar to the parental lineages. Little is known about the ORF8:Q18* mutation found in the XJ lineage, its impact on infection remains to be elucidated. Further studies addressing transmissibility, disease severity and immune evasion are needed to assess the public health impact that the recombinant may have.

We did not identify an index case, leaving the origin of the XJ recombinant unresolved. The presence of both of the potential parental lineages in the geographical region where the XJ lineage was first detected, suggests that the recombination event may have occurred in the Länsi-Pohja region or in its proximity. However, since genomes identical to these potential parental sequences have also been detected elsewhere, we cannot exclude the possibility of introduction from another country.

## Conclusion

The recombinant XJ virus has established continuous community transmission in north-western Finland and has dispersed to other parts of Finland and abroad. Global genomic variant surveillance plays a pivotal role in timely tracking of variants and for our increased understanding of the evolutionary dynamics of the pandemic SARS-CoV-2 virus.
